# A Novel Solution to Enhance the Oxidative and Physical Properties of Cookies Using Maltodextrin-Based Nano-Sized Oils as a Fat Substitute

**DOI:** 10.1021/acsomega.5c01200

**Published:** 2025-05-28

**Authors:** Raciye Meral, Mehmet Mustafa Eki̇N, Zafer Ceylan, Aslıhan Alav, Erol Kına

**Affiliations:** † Department of Food Engineering Faculty of Engineering, 53000 Van Yüzüncü Yıl University, Van 65080, Turkiye; ‡ Özalp Vocational School, Food Technology Program, Van Yuzuncu Yıl University, Van 65080, Turkiye; § Faculty of Science, Department of Molecular Biology and Genetics Kutlubey Campus, Bartin University, Kutlubeyyazıcılar Village, Bartin 74110, Turkiye; ∥ Van Yüzüncü Yıl University, Institute of Science, Van 65080, Turkiye; ⊥ Computer Programming Program, Özalp Vocational School, Van Yüzüncü Yıl University, Van 65100, Turkiye; # Innovan Entrepreneurship Centre, Van 65080, Turkiye

## Abstract

This study investigated the effects of maltodextrin-based nanoemulsions as fat substitutes in cookies, focusing on the oxidative stability and physical properties. Full-fat cookies (control, C) and 50% fat-reduced cookies with nanoemulsions (FC) were produced. The addition of nanoemulsions increased the cookie diameter from 46.3 mm (control) to 56.1 mm and reduced the thickness, resulting in a desirable texture. Initial hardness values (30.3 and 45.8 N) were lower in nanoemulsion samples and remained reduced over a 90 day storage period. Black cumin oil-loaded nanoemulsions provided the lowest peroxide values (1.7, 2.7, and 2.4 mequiv O_2_/kg), maintaining oxidative stability during storage. Final free fatty acid (FFA) values ranged from 0.23% to 0.44% after storage. Thiobarbituric acid (TBA) values indicated slower lipid oxidation, with values ranging from 1.47 to 2.51 mg MDA/kg on day 0 and increasing to a maximum of 4.13 mg MDA/kg by day 90 in fat-reduced cookies. Among the tested formulations, nanoemulsions enriched with black cumin oil demonstrated the highest effectiveness, yielding enhanced oxidative stability and improved quality characteristics. This study presents an innovative strategy by utilizing maltodextrin-based nanoemulsions containing naturally antioxidant-rich oils as fat replacers, offering a clean-label alternative to improve the oxidative resilience and physical quality of cookies.

## Graphical abstract

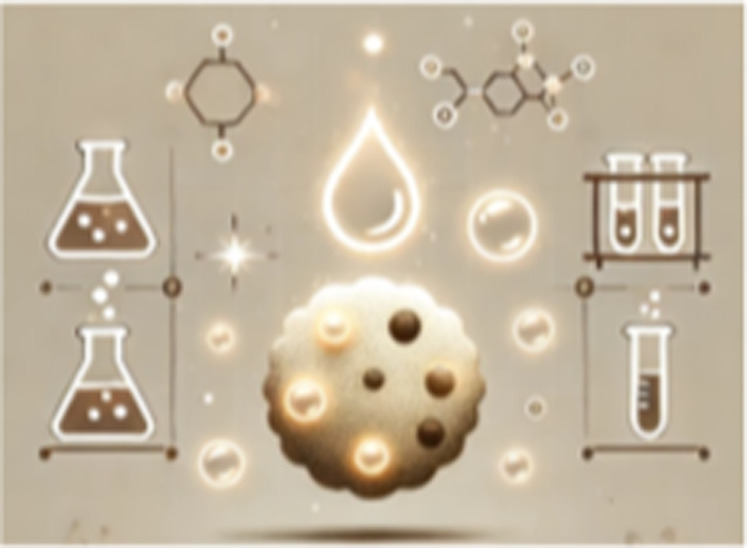

## 1 Introduction

Cookies are one of the most popular bakery-finished products consumed worldwide that is made from cereal. Such a large amount of demand is the result of affordability, high flavor, and long shelf life.^1,2^ Cookies are characterized by low moisture and high fat and sugar content. High fat and sugar levels both contribute significantly to the texture and the overall acceptability of cookies. Consumer evaluations consistently show that fats improve flavor, texture, and acceptance.^3^ Shortening is a type of fat that is used to make bakery products, such as cakes and cookies. It is solid fat and includes 63% saturated fat.^4^ In terms of sensory and physical quality, fat is the most crucial component for high consumer acceptability. As they compete with water to cover the flour surface during kneading, fats fulfill the role of a plasticizer by preventing the formation of an excessive gluten network. Limiting the formation of gluten provides a soft and crunchy feel^5,6^

However, recent trends toward healthier diets have focused on fat reduction strategies, not only to support nutritional goals but also to reduce the oxidative potential in such products. Due to the fatty acid profile in cookies, they are susceptible to oxidation, which can impact their shelf life and flavor. Therefore, reducing fat content may also contribute to improved oxidative stability in these bakery products.^7^

Given the fast-rising prevalence of overweight and obesity—once considered issues primarily in high-income countries but now increasingly affecting urban areas in low- and middle-income countries—there is a growing interest in creating healthier food alternatives. Accordingly, food manufacturing and the bakery food industry have started exploring new recipes that balance consumer demand for healthier options without compromising quality.^8^ ^,^ ^9^

In this sense, protein- and carbohydrate-based fat substitutes are used instead of fat in bakery products. However, with the reduction of fat, the acceptability of the cookie by the consumer also decreases and undesirable problems such as reduced crispness and increased hardness occur in cookies. In this context, it is thought that the problems related to fat reduction in cookies can be eliminated by using nanomaterials. When compared to their bulk counterparts, nanomaterials have advantages including strength, reactivity, high surface area, less quantity, and greater efficiency.^2,10–14^ The use of nanoemulsions, which are now prevalent in many industries, has begun to grow in the food sector as a result of their benefits, such as increased functionality and optical transparency. In addition to protecting the active ingredients from external factors like heat, light, and oxidation, nanoemulsions also make it easier to distribute water-insoluble ingredients and increase the surface area due to their nanosize.^15–17^ Our previous study demonstrated that nano-oil can be used in cookie^18^ production without compromising quality attributes, such as flavor, texture, crispness, and spread ratio.^2^ Another study also suggested that nano-oil might be an effective fat replacer in baked products with reduced fat content.^19^ The goal of the present study was to produce fat-reduced cookies that could have limited oxidation levels and better textural properties using a nanoemulsion fabricated from sesame oil, grape seed oil, coconut oil, and black cumin oil. In this respect, the final aim was to improve the functional characteristics of these cookies with the present technological application.

This study introduces a novel approach by employing maltodextrin-based nanoemulsions loaded with natural antioxidant-rich oils as fat substitutes in cookies. Unlike previous studies that used conventional fat replacers, this method utilizes nanoemulsions to simultaneously enhance the oxidative stability and improve physical properties. This innovative approach not only provides a clean-label solution but also offers a potential solution for improving product stability and maintaining desirable sensory properties, addressing the texture challenges commonly observed in fat-reduced cookies. Additionally, the use of antioxidant-rich oils within the nanoemulsion matrix enhances oxidative stability, potentially extending the product’s shelf life without the need for synthetic preservatives. This dual-action strategy represents a promising advancement in the development of healthier, more stable bakery products.

## 2 Material and Methods

### 2.1 Material

Sesame oil, grape seed oil, coconut oil, and black cumin oil were obtained from Arapaş Arifolu Marketing Distribution and Trade Inc. in Istanbul, Türkiye. They were utilized in the manufacture of nanoemulsion. The Meram Flour Factory provided the flour (Konya, Turkiye). Maltodextrin and Tween 20 were acquired from Sigma-Aldrich (St. Louis, MO, USA).

### 2.2 Nanoemulsion Preparation

Sesame oil, grape seed oil, coconut oil, and black cumin oil were employed as oil phases in the fabrication of nanoemulsions (NEs). The coarse emulsion was created by using an Ultra Turrax T25 (IKA, Germany) to blend 10 mL of maltodextrin (1%, w/v) and 0.1 g of Tween 20 with the oil phase (1 g oil) for 5 min at a speed of 16000 rpm.^17,19^ Each coarse emulsion was then subjected to ultrasonic homogenization for 15 min at a 60% amplitude to reduce particle size^20^. Nanoemulsion samples were coded as SO (sesame-oil-loaded nanoemulsions), GSO (grape seed-oil-loaded nanoemulsions), BSO (black cumin-oil-loaded nanoemulsions), and CNO (coconut oil-loaded nanoemulsions).

### 2.3 Characterization of Nanoemulsions

Utilizing cryogenic transmission electron microscopy (Cryo-TEM), the morphology of the nanoemulsion was studied (Hitachi HT7800, Japan). On a copper TEM grid, 5 μL of diluted sample was spread out. The samples were swiftly submerged into liquid ethane at −165 °C after around 3 to 5 h. The nanoemulsins (NEs) were subsequently moved to a cryogenic sample container for TEM imaging at −174 °C. The apparatus was used at 120 kV and has a magnification range of 6300–12,500 20. Differential scanning calorimetry (DSC) analysis was conducted to investigate the thermal properties of the nanoemulsion samples. The measurements were performed using a DSC instrument (Bakher, Germany) under controlled conditions. Approximately 2–5 mg of the sample was placed in hermetic aluminum pans to ensure a sealed environment, preventing evaporation or oxidation during the thermal analysis. The pans were tightly sealed to maintain the sample integrity throughout the measurement process.

The thermal behavior was analyzed over a temperature range of 25–350 °C with a constant heating rate of 10 °C min^–1^. An empty hermetic aluminum pan was used as the reference to ensure accurate baseline correction. The measurements were carried out in an inert nitrogen atmosphere with a flow rate of 50 mL min^–1^, ensuring a stable and consistent environment throughout the analysis.

### 2.4 Cookie Preparation

In the investigation, samples for the 50% fat reduction (based on flour weight) without nanoemulsion (FC) were also created in addition to the samples for the control group (C). Various samples incorporating 20% nanoemulsion, calculated based on flour weight, were prepared to evaluate the effect of fat reduction on cookie formulation (Table 1). The codes for the produced cookies are as follows: C: control cookies, FC: fat-reduced cookies without nanoemulsion, GS-C: grape seed oil-loaded nanoemulsion-containing cookies, SO–C: sesame oil-loaded nanoemulsion-containing cookies, CO–C: coconut oil-loaded nanoemulsion-containing cookies, and BC-C: black cumin oil-loaded nanoemulsion-containing cookies.

**Table 1:** Cookie Formulation^a^

	cookies^a^					
ingredients	C	FC	GS-C	SO–C	CO–C	BC–C
wheat flour (g)	100.0	100.0	100.0	100.0	100.0	100.0
sugar (g)	40.0	40.0	40.0	40.0	40.0	40.0
shortening (g)	40.0	20.0	20.0	20.0	20.0	20.0
nanoemulsion (g)	0.0	0.0	20.0	20.0	20.0	20.0
water (g)	22.0	22.0	4.0	4.0	4.0	4.0
sodium bicarbonate (g)	0.75	0.75	0.75	0.75	0.75	0.75
ammonium bicarbonate (g)	0.25	0.25	0.25	0.25	0.25	0.25
corn syrup (g)	1.5	1.5	1.5	1.5	1.5	1.5
salt (g)	1.0	1.0	1.0	1.0	1.0	1.0
vanilla (g)	1.0	1.0	1.0	1.0	1.0	1.0

a C: Control cookies, FC: fat-reduced cookies without nanoemulsion, GS-C: grape seed oil-loaded nanoemulsion-containing cookies, SO–C: sesame oil-loaded nanoemulsion-containing cookies, CO–C: coconut oil-loaded nanoemulsion-containing cookies, and BC-C: black cumin oil-loaded nanoemulsion-containing cookies.

The cookie dough was prepared by referring to Meral et al. (2022).^21^ Shortening, sugar, salt, vanilla, and sodium bicarbonate were mixed for 3 min at 60 rpm. After another minute of vigorous stirring, water, ammonium bicarbonate, and high-fructose corn syrup were added to the mixture. Wheat flour was finally added to the mixture, and the mixture was combined for 2 min at 60 rpm to complete the dough preparation process. In order to shape the cookie dough, the methods recommended by AACC (10–50.05) were used.^22^ The cookies were shaped into uniform discs with an initial diameter of 6 cm before further processing. The cookies were stored for 90 days in airtight containers at room temperature (approximately 20–25 °C) in a dry, dark environment to minimize exposure to humidity and light, which could impact the texture and shelf life. These conditions were maintained consistently to ensure the stability of the product’s sensory and physical qualities throughout the storage period.

### 2.5 Physical Properties

A TA.XTPlus Texture analyzer (Stable Micro Systems, UK) measured the hardness of the cookies. The force required to break the cookie (cookie hardness, N) was determined (load cell: 5 kg, pretest speed: 1.0 mm/s, test speed: 5.0 mm/s, post-test speed: 10.0 mm/s, distance: 10 mm, trigger force: 50 g). A Minolta Chroma Meter was used to determine the color of the baked cookies using L*, a*, and b* values.^23^

### 2.6 Extraction of Oil

The technique outlined by Bakkalbaşi et al. (2012)^24^ was used to extract the oil. Oil extraction from the cookies was carried out using a cold extraction method. The milled sample was combined with ten times the amount of hexane. This mixture was homogenized with a homogenizer (Heidolph, Germany) at 10,000 rpm for 30 s. After homogenization, the mixture was then transferred to an extraction flask under a nitrogen atmosphere and shaken on a circular shaker at 200 rpm in the dark at ambient temperature for 2 h. The mixture was then filtered. This extraction process was repeated twice. The layers of solvent obtained from the combined filtrates were evaporated by using a rotary vacuum evaporator.

### 2.7 Free Fatty Acid (FFA) and Peroxide Value (PV)

FFA measures the breakdown of fats into free fatty acids, indicating the quality and freshness. PV measures initial oxidation products (peroxides), assessing oxidative stability. Oil was extracted. The method of Hoffpauir et al., (1947),^25^ a titration approach in which % FFA is calculated as oleic acid, was followed. PV was calculated using the AOCS Official Method.

FFA content was determined following the official titration method. For this purpose, 2 g of the sample was weighed into a 250 mL Erlenmeyer flask. Then, 25 mL of a neutralized ethanol solution was added, and the mixture was heated to 60 °C while stirring gently. After the mixture was cooled to room temperature, 2–3 drops of phenolphthalein indicator were added to the solution. The sample was titrated with 0.1 N sodium hydroxide (NaOH) solution until a persistent light pink color was observed. The FFA content was calculated by using the following formula

$$FFA\;\left(\%\right)=\frac{V\times N\times 28.2}{W}$$

where

- *V* = Volume of NaOH solution used (mL)
- *N* = Normality of NaOH solution
- *W* = Weight of the sample (g)
- 28.2 = Conversion factor for oleic acid

The peroxide value (PV) was determined based on the iodometric titration method. Approximately 5 g of the sample was weighed in a 250 mL Erlenmeyer flask. Then, 30 mL of acetic acid-chloroform solution (3:2 ratio) was added, followed by 0.5 mL of a saturated potassium iodide (KI) solution. The flask was shaken vigorously for 1 min and then allowed to stand in the dark for 5 min. Afterward, 30 mL of distilled water was added, and the liberated iodine was titrated with 0.01 N sodium thiosulfate (Na_2_S_2_O_3_) solution using starch as an indicator until the blue color disappeared. The PV was calculated by using the following formula

$$PV=\frac{\left(V-V0\right)\times N\times 100}{W}$$

Results were expressed as milliequivalent O_2_ (meq O_2_/kg)

where.

- *V* = Volume of Na_2_S_2_O_3_ solution used for the sample (mL)
- *V*_0_ = Volume of Na_2_S_2_O_3_ solution used for the blank (mL)
- *N* = Normality of Na_2_S_2_O_3_ solution
- *W* = Weight of the sample (g)

In this study, the peroxide value (PV) and free fatty acid (FFA) contents were calculated using Python programming. A custom Python code developed by Erol KINA was employed to implement the required titration formulas, enabling fast, accurate, and efficient analysis of the data.

### 2.8 Analysis of Thiobarbituric Acid (TBA)

The flask was filled with 10 g of homogenized samples, 4N 2.5 mL of HCl, and 97.5 mL of distilled water before being boiled to extract the distillate. Test tubes were filled with 5 mL of distillate and 5 mL of TBA reagent, and then the tubes were sealed, agitated, and heated in a beaker of boiling water for 35 min. Using a spectrophotometer, the absorbance was measured at 538 nm against the blank after the tubes were cooled in water for 10 min (Shimadzu-UV mini 1240, Kyoto-Japan). The total number of samples’ TBA findings came out to mg of malondialdehyde per kg (mg MDA/kg).

### 2.9 Statistical Analysis

An ANOVA test was performed to examine the differences across groups. This analysis was conducted by using Python-based tools. These tools are often preferred in data science projects due to their flexible structures. The integration of Python tools in statistical analysis can facilitate the handling of complex data patterns and enhance the reliability of results. AI-based methods can play a supportive role in the analysis process by assisting with data preprocessing, automating modeling tasks, and enhancing data visualization, particularly when dealing with large and complex data sets.^26–30^ Since the ANOVA results indicated significant differences among groups, posthoc comparisons were conducted using the Tukey HSD test to determine which groups differed from each other.^18,31^

## 3 Results and Discussion

### 3.1 Cryo-TEM

Figure 1 shows TEM images of the nanoemulsions. It can be seen from the images that each nanoemulsion had different shapes. The BSO nanoemulsion loaded with black cumin oil contained spherical-shaped particles, while CNO and SO contained particles in the form of spherical filaments. The GSO nanoemulsion was seen as spherical, polygonal, and rod-shaped particles. It was expected that there would be differences due to the composition of the oils and the drying processes performed before and during the TEM analysis. TEM images revealed that nanoparticles ranging in size from 200 to 300 nm were produced. Ekin et al. (2019)^2^ noted that the presence of nanosized particles was demonstrated with TEM and DLS. The used material can play an important role to obtain lower-sized material. In this regard, wheat germ oil nanoemulsions had 114.7 nm average size as described by Ceylan et al.^15^

**Figure 1:**
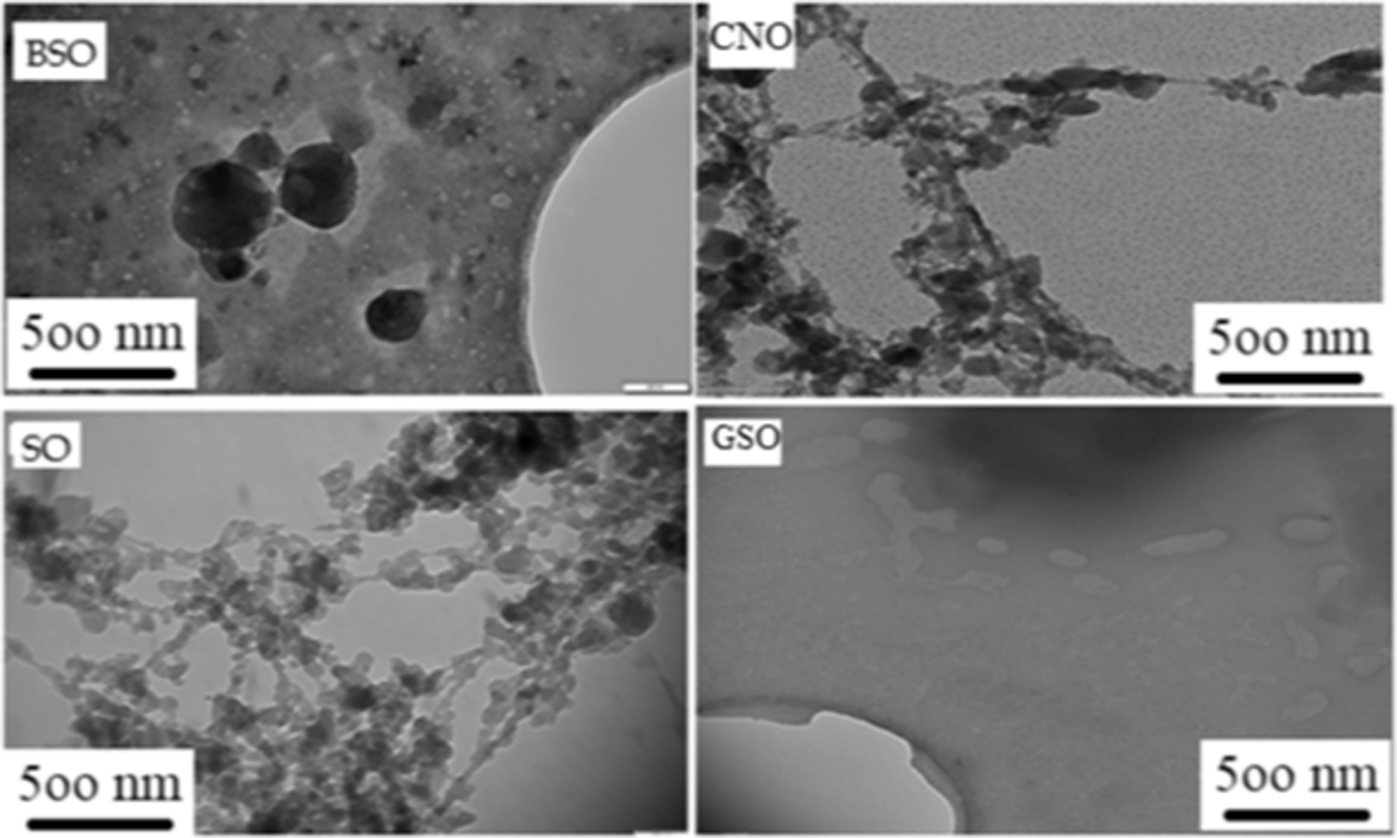
Cryo-TEM images of nanoemulsion.

### 3.2 Differential Scanning Calorimetry

The thermal properties of the nanoemulsion samples were determined by DSC and the obtained thermograms are given in Figure 2. The endothermic peaks indicating melting were determined in all nanoemulsion samples. The onset points of peaks were determined as 35 °C for CNO, 41.35 °C for BSO, 70 °C for GSO, and 105 °C for SO. The melting event of CNO occurred in a lower range than the other samples, which indicates that the melting of CNO was much more rapid. Generally, oils with a high level of saturated fatty acids have greater melt crystallization temperatures than oils with a low content of saturated fatty acids. Coconut oil has an iodine value of 9.37, meaning it contains a lot of saturated fatty acids.^32^ It could be seen from DSC thermograms that the thermal resistance of SO samples is higher than that of other nanoemulsions. Suksuwan et al. (2021)^33^ determined an endothermic peak indicating melting at 24.5 °C in the DSC thermograms of lipid nanoparticles they prepared using black cumin oil. In the same study, an endothermic peak was determined at 23.1 °C in a DSC thermogram of pure black cumin oil. The results demonstrated that the thermal resistance of the nanoparticle form of black cumin oil is better than that of pure black cumin oil. Tan and Che Man^32^ examined the thermal behavior of coconut oil with DSC and they found that the melt onset temperature for coconut oil was 13 °C. According to Sivakanthan et al.,^34^ sesame and coconut oils had melting points of 25.07 ± 0.12 and 4.1 ± 0.17 °C, respectively. Considering the melting points of the oils, it was observed that the melting temperatures of the oils encapsulated in the nanoemulsion were increased. The observed increase in the melting temperature can be attributed to the encapsulation of the oils within the nanoemulsion matrix. Encapsulation restricts the mobility of the oil molecules, creating a more stable structure that requires higher energy to transition from a solid to a liquid state.

**Figure 2:**
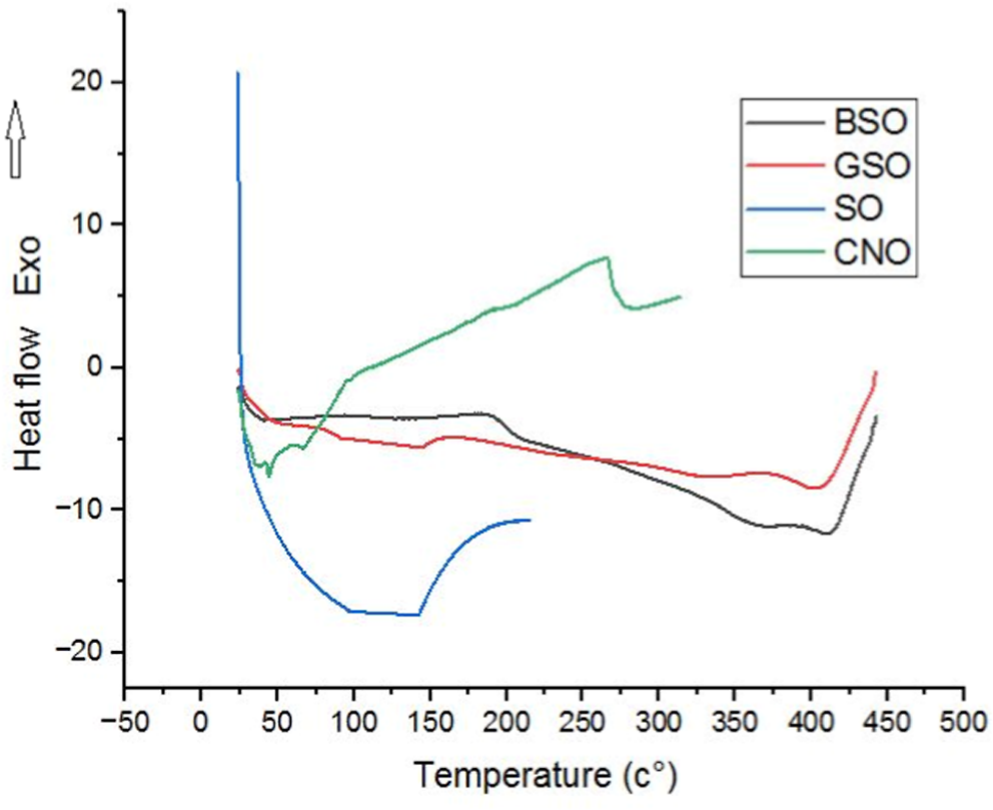
DSC thermograms of nanoemulsion.

### 3.3 Diameter and Thickness

The diameter and thickness values of the cookies are given in Table 2. The diameter of cookies containing nanoemulsions varied between 55.76 and 56.13 mm. This value was found as 46.33 and 48.53 mm, respectively, in the control (C) and FC cookies not containing nanoemulsions. The diameter were significantly increased with the addition of nanoemulsion (*p* < 0.05). While the highest diameter was obtained in CO–C, this sample was followed by BC-C, SO–C, and GS-C cookies (*p* > 0.05). The thickness values of the cookies varied between 6.00 and 7.52 mm. The lowest thickness was determined in GS-C containing grape seed oil-loaded nanoemulsion, and the highest thickness values were determined in the control group. The addition of nanoemulsion in the formulation had a reducing effect on the thickness value. In a study in which apricot kernel flour was used as a fat substitute in biscuits, the diameter was decreased from 7.60 cm (control) to 7.23 cm, and the thickness was increased from 1.02 to 1.21 cm.^35^ In another study investigating the possibility of lotus root flour as a fat replacer in biscuits, the diameter in the control group was 4.45 cm, and the thickness was 0.89 cm. With 25% fat replacement, the diameter was 4.31 cm and the spreading rate was determined 4.17 ^36^. Paciulli et al. (2020)^37^ determined that the diameter of the biscuits was decreased and the thickness was increased with increasing the amount of fat substitute. Conforti et al. (1997)^38^ reported that fat reduction in cookies resulted in a denser biscuit texture, and the width of the biscuits was significantly reduced.

**Table 2:** Physical Properties of Cookies

cookies	diameter	thickness	*L* ^a^	*a* ^a^	*b* ^a^
C	46.33 ± 0.95^c^	7.52 ± 0.04^a^	68.74 ± 4.49^ab^	3.75 ± 1.83^a^	29.83 ± 1.44^b^
FC	48.53 ± 0.29^b^	7.43 ± 0.00^b^	74.71 ± 2.11^a^	3.90 ± 2.20^a^	29.39 ± 2.95^b^
BC-C	56.07 ± 0.17^a^	6.08 ± 0.00^d^	66.40 ± 1.01^b^	6.53 ± 0.22^a^	32.12 ± 0.40^ab^
CO–C	56.13 ± 0.19^a^	6.68 ± 0.00^c^	66.20 ± 0.69^b^	6.53 ± 0.53^a^	33.27 ± 0.17^ab^
SO–C	55.76 ± 0.07^a^	6.08 ± 0.00^d^	67.56 ± 2.77^b^	6.03 ± 1.46^a^	34.24 ± 1.19^a^
GS-C	56.00 ± 0.11^a^	6.00 ± 0.00^e^	67.30 ± 1.27^b^	5.25 ± 1.29^a^	31.82 ± 0.37^ab^

a C: Control cookies, FC: Fat-reduced cookies without nanoemulsion, GS-C: Grape seed oil-loaded nanoemulsion-containing cookies, SO–C: Sesame oil-loaded nanoemulsion-containing cookies, CO–C: Coconut oil-loaded nanoemulsion-containing cookies, and BC-C: Black cumin oil-loaded nanoemulsion-containing cookies The letters (a, b, c, d) indicate statistical differences between the samples. Within the same column, values that share the same letter are not significantly different from each other, while values with different letters indicate significant differences (*p* < 0.05).

A desirable cookie was defined as a cookie with a large diameter value and a low thickness value (Guttieri et al., 2008),^39^ and higher diameter and lower thickness were obtained with the increase of fat content in cookies. This effect may be attributed to the increased mobility of fat molecules as the fat melts during baking, leading to an increase in the cookie diameter. In a significant part of the studies on the effects of fat reduction on the quality of cookies, it was revealed that there was a decrease in cookie diameter with fat reduction. This study’s findings differed from those of other researchers, who reported a reduction in cookie diameter with decreased fat content. Cookies with a 50% fat reduction and 2 g of nanosized oil in place of shortening showed greater diameter and lower thickness values compared to the control and FC samples. The increased diameter observed in cookies containing nanoemulsions, despite their lower fat content, could be attributed to the unique properties of nanosized oil droplets. The significantly reduced size of these droplets leads to an expanded surface area, allowing the oil particles to interact more effectively with the dough matrix. This enhanced interaction ensured a more uniform structural composition by distributing the oil evenly throughout the dough. In summary, the application of nanotechnology through nanoemulsions improves the dough’s capacity to expand, resulting in cookies with a larger diameter even at reduced fat levels. This approach highlights the potential of nanoemulsions to enhance product performance.

### 3.4 Surface Cracks

A crucial quality criterion for cookies is their exterior appearance.^40^ In this context, ″islands″ refer to distinct, uniform patches or regions visible on the cookie surface, a term commonly used in cereal technology to describe similar patterns in baked goods. As shown in Figure 3, reducing the fat content by 50% increased the number of surface cracks.^5^ In cookies containing the nanoemulsion, islands were uniform and medium-sized, while in full-fat cookies, these islands appeared smaller and more concentrated.

**Figure 3:**
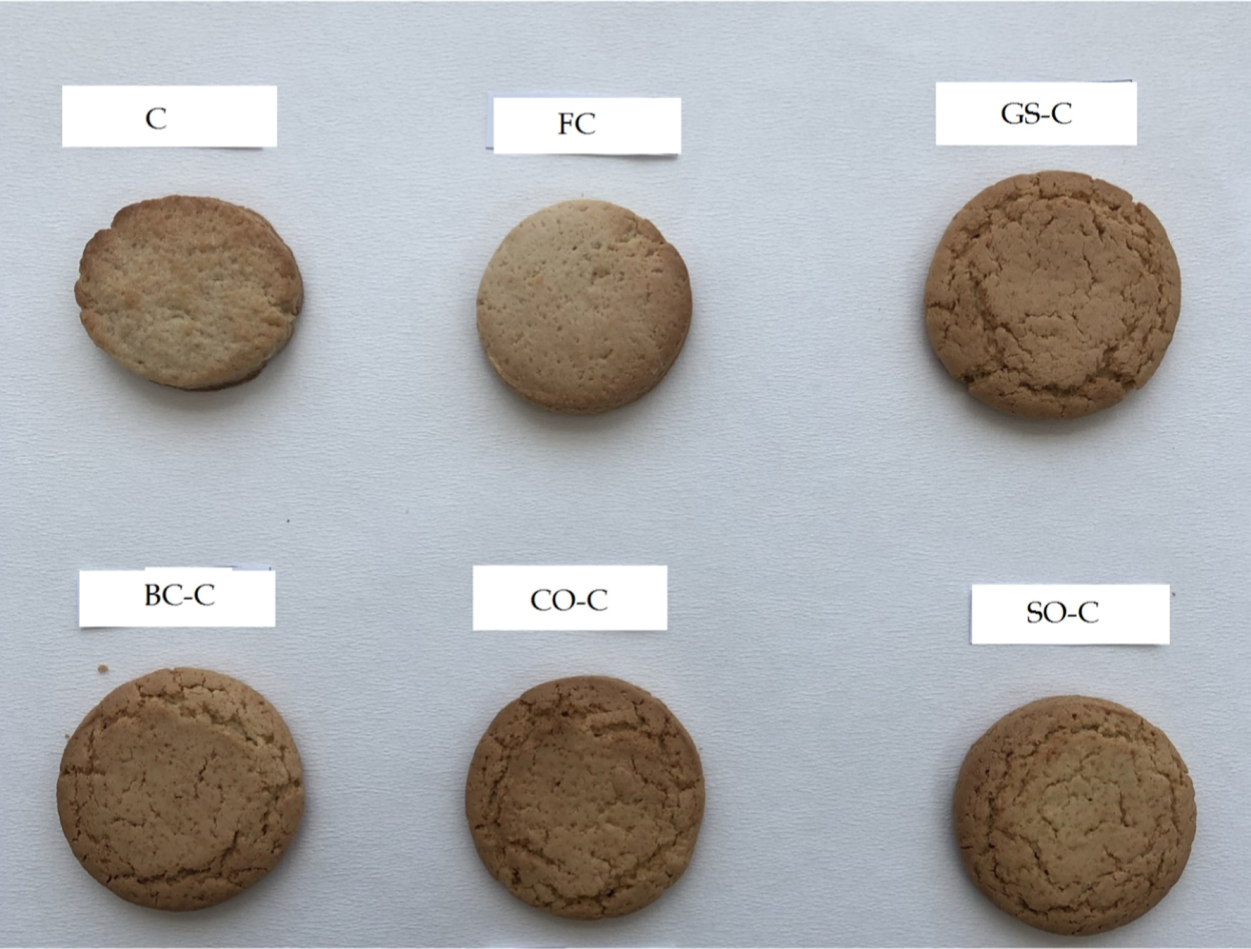
Appearance of Cookies.

### 3.5 Color

Color is one of the criteria determining the purchase intention of consumers. In this study, the effect of nanoemulsion addition on the color values of cookies was investigated, and the results are given in Table 2. L* values of samples varied between 66.20 and 74.71, a* values were between 3.75 and 6.53, and b* values were between 29.39 and 34.24. L* and b* values were significantly affected by nanoemulsion addition (*p* < 0.05), and the effect of nanoemulsion addition on a* value was insignificant (*p* > 0.05). The L* value, which was 68.74 in the control group, increased to 74.71 in the fat-reduced control sample (FC), and this value showed a decreasing trend in the samples containing the nanoemulsion. According to the statistical analysis, no difference was found between the control group samples and the samples containing nanoemulsion (*p* > 0.05), but a significant difference was found between the L* values of the FC and the samples containing the nanoemulsion (*p* < 0.05).

In the cookie samples, the highest a* score was determined in the BC-C samples containing nanoemulsions prepared using black cumin oil, and it was revealed that the addition of nanoemulsion to the formulation did not have a significant effect on the a* values of the cookies (*p* > 0.05). The a* value of all biscuits produced was on a positive scale. The control (C) and fat-free control (FC) group samples had the lowest b* values, and the yellowness value increased with the addition of nanoemulsion. Yalçın & Maden (2017)^41^ used yellow poppy seeds as a fat replacer in biscuits and as the oil substitute increased, the L* scores were decreased from 71.12 to 67.49. The a* and b* values increased from 12.42 to 13.73, from 30.60 to 32.12, respectively. In another study in which lotus root flower was used as a fat substitute, the surface color lightness (L*) and yellowness (b*) values of the biscuits were decreased, and the redness (a*) values were increased, when the substitution rate was increased.^36^ As could be understood from the literature data, the brightness, redness, and yellowness values of biscuits could be varied, depending on the characteristics of the substituents.

### 3.6 Hardness

To determine the hardness values of the cookie samples during storage, texture analysis was performed on the zeroth, 45th, and 90th days of storage, and the values obtained are presented in Figure 4. The hardness of the cookies were found to be between 30.30 and 45.81 N on the first day of cookie production. On the initial day, control and nanoemulsion-free and reduced fat (FC) cookie samples were the hardest cookies. The hardnesses of the cookies containing the nanoemulsion were similar to each other and were lower than the control group. The lowest hardness was obtained from the cookie (30.30 N) prepared using sesame oil nanoemulsion. On the 45th day of storage, the hardness of the control group sample was determined as 47.01 N, while this value was determined as 59.22, 41.26, 36.10, 46.55, and 29.20 N for FC, BC-C, CN–C, SO–C, and GS-C samples, respectively. As a result of the analysis made on the 45th day of storage, the hardness of the cookies increased as expected. The hardness of the samples containing the nanoemulsion was lower than that of the control group at the end of the 45th day of storage.

**Figure 4:**
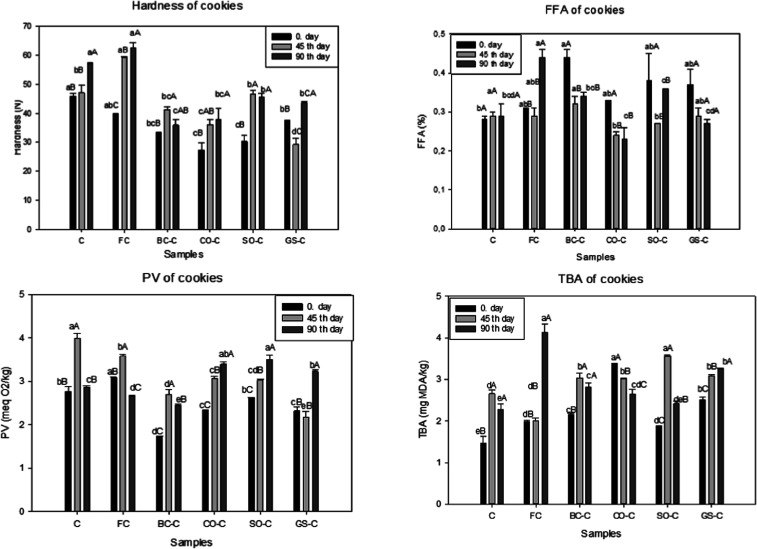
Changes in hardness, FFA, PV, and TBA values during storage. Lowercase letters indicate differences among samples on the same storage day (*p* < 0.05). Uppercase letters represent differences within the same sample across different storage days (*p* < 0.05). Values are presented as mean ± standard deviation.

At the end of the 90th day, the hardness was changed between 35.91 (CN) and 62.56 (FC) N. When compared to the initial day, an increase in the hardness value of all cookie samples was observed at the end of 90 days. When the first day and last day data were evaluated, the highest increase in hardness value was detected in the FC sample with an increase of 57.6%, while the least increase was detected in cookies prepared with black cumin oil nanoemulsion (increase: 7.8%). In a study, 10%, 20, 30, and 40% defatted tomato seed flour was used instead of fat in the cookie formulation, and the hardness value of the cookie increased as the substitution rate was increased. Yashini et al. (2021)^42^ reported that the hardness of the control was 38.37 N; the hardness value of cookies was increased to 48.30, 58.75, 61.67, and 73.25 N with increased fat reduction. Paciulli et al. (2020)^37^ used an emulsion-filled gel (EFG) as a fat replacer. They reported that biscuit hardness values were increased with the increase of the substitution ratio. It was reported by Krystyjan et al. (2015)^43^ and Santiago-García et al. (2017)^44^ that cookie hardness increased with the reduction of fat content. This effect can be explained by the role of fat as a tenderizing agent in the dough. Fat coats the flour particles and competes with water for gluten development, reducing the formation of a strong gluten network. When the fat is reduced, the gluten structure becomes more cohesive, leading to increased hardness in the final baked product.

Hardness was increased depending on the increase in fat reduction levels. The hardness value of the cookies prepared with nanoemulsions was lower than the hardness value of the control sample. This effect may be associated with the broader coverage of nanosized oil particles on the dough surface, which is characteristic of nanotechnology. Fat reduction is one of the biggest problems encountered in the selection of fat replacers and complicates the fat reduction process. The data obtained from this study showed that the use of nanoemulsions as a fat substitute in cookie production would be beneficial in terms of solving the hardness problem caused by fat reduction in cookies.

### 3.7 Free Fatty Acid

The FFA results are given in Figure 4. The FFA of the cookie samples on the zeroth day varied between 0.28 and 044%. At the end of storage, FFA was changed in the range 0.23 (BC–C)-0.44% (FC). In a study in which grape pomace extract was used in the cookie formula, it was reported that the grape pomace extract was more effective in reducing the FFA than the samples prepared with control and BHA.^45^ Aksoylu et al. (2015)^46^ produced biscuits using blueberry, defatted grape seed flour, and defatted poppy seeds. The FFA of control biscuits was increased from 0.36% to 0.49% after 5 months of storage. While the FFA value was found to be 0.33% in biscuits prepared with grape seed flour, it increased to 0.45% in the third month of storage and decreased to 0.38% at the end of the fifth month.

In the present study, on the initial day of storage, the FFA of the control and FC cookies whose fat was reduced by 50% was lower than that of the cookies containing nanoemulsion. The result could be explained in several ways.

- Lipid oxidation leads to the formation of conjugated diene systems at the alpha methyl carbons of unsaturated fatty acids due to hydrogen loss. These conjugated dienes then break down, producing peroxides, aldehydes, and ketones. Acidity results from the release of fatty acids from triglycerides during hydrolytic rancidity. According to Lopez et al. (2022), one possible explanation for low FFA content is that the rate of peroxide and decomposition product formation exceeds the rate of free fatty acid production.^47^ As can be seen in Figure 4, the PV of the control and FC group on day 0 is higher than the other samples.
- Synthetic phenolic antioxidants such as butylated hydroxy toluene (BHT) and butylated hydroxy anisidine (BHA) are added to the shortenings. In the treatment using BHT, the mechanism halts the oxidation propagation chain through electron transfer and proton donation.^48^

On the 45th and 90th days of storage, a significant decrease was observed in the FFA of the cookie containing nanoemulsion compared to the initial day (*p* < 0.05). On the 90th day of storage, the FFA of the CO–C and GS-C samples were found to be lower than the control group. The highest FFA was determined in the FC samples after 90 days of storage. On the 90th day, the FFA of the FC sample increased 1.5 times compared to the initial day. As a result, FFA exhibited a fluctuation pattern throughout storage. During 90 days of storage, the FFA of GS-C and BC-C was lower than those of control samples, while the FFA values of the CO– C, and SO–C were lower than the FC whose fat reduced by 50%. On the 90th storage day, the FFA of the samples containing nanoemulsions were lower compared to the zeroth day. This result could be attributed to the presence of antioxidant compounds in the oils contained within the nanoemulsions. Natural antioxidants may act through mechanisms similar to those of synthetic antioxidants or may accelerate termination reactions. Essential oils contain antioxidant molecules that work through various mechanisms. Phenolic antioxidants, in particular, function by interrupting the propagation of oxidation and are referred to as “chain-breaking antioxidants”.^48^ The decrease in FFA levels at the end of 90 days, compared to the beginning could also be attributed to the slow and controlled release of antioxidant compounds in the nanoemulsions, which effectively helped to prevent hydrolytic rancidity and the release of free fatty acids.

### 3.8 Peroxide Value (PV)

The PV of the oils extracted from the cookies is presented in Figure 4. At the beginning of storage, the peroxide values (PV) ranged from 1.72 mequiv O_2_/kg (BC–C) to 3.09 mequiv O_2_/kg (FC). On the 45th and 90th storage days, PV values ranged between 2.18 mequiv O_2_/kg (GS-C) and 3.99 mequiv O_2_/kg (C) and 2.87 mequiv O_2_/kg (C) and 3.23 mequiv O_2_/kg (GS-C), respectively.

In previous studies, different PV values have been reported for cookies. For instance, in low-fat biscuits containing polydextrose and guar gum, the PV ranged from 1.44 to 5.11 mequiv O_2_/kg and 1.24 to 3.43 mequiv O_2_/kg, respectively, during a 90 day storage period. Findings align with the PV values obtained in our study,^49^ indicating that the observed results fall within the typical range reported in the literature. Significant differences (*p* < 0.05) were observed among the PV values of cookies in the present study.

On the initial and 45th storage days, the control (C) and fat-reduced control (FC) cookies showed higher PV values compared to the other samples. The lowest PV was observed in BC-C samples prepared with black cumin oil-loaded nanoemulsions. This suggests that the antioxidant properties of black cumin oil, particularly its γ-tocopherol content, effectively reduced lipid oxidation. Furthermore, the lower PV in nanoemulsion-prepared cookies highlights their protective role against autoxidation during the baking process. This finding aligns with earlier studies, which suggested that antioxidant compounds in dough formulations can limit oxidation during baking.

For example, Sultan et al. (2012)^50^ reduced shortening levels by 1–5% (T1–T5) and replaced them with black cumin oil. The PV decreased progressively with higher black cumin oil levels, reaching 0.213 mequiv O_2_/kg for T5 compared to 0.288 mequiv O_2_/kg for the control. The decline in PV was attributed to the high α-tocopherol content in black cumin oil. Similarly, in the current study, the cookies prepared with black cumin oil nanoemulsions (BC–C) consistently demonstrated the lowest PV throughout storage.

On the 90th day, a decrease in PV values was observed for the C, FC, and BC-C samples compared to the 45th day. This reduction is likely due to the formation of secondary oxidation products, as oxidation progresses from the primary to the secondary stage during extended storage.^51^ As shown in Figure 4, the PV of cookies containing nanoemulsions (except BC-C) was higher than the control (C) on the 90th day. This difference can be attributed to the higher unsaturated fat content and the reduction in the antioxidant-containing shortening in these samples. Additionally, the nano-oil’s expanded surface area may have increased its susceptibility to oxidation. However, further studies are needed to validate this hypothesis.

Feng et al. (2020)^52^ demonstrated that tocopherol-loaded nanoemulsions (400–450 nm particle size) were more effective in reducing lipid oxidation in fish sausages compared to coarse emulsions (4000–4500 nm). On the eighth, 12th, and 16th storage days, samples with coarse emulsions had significantly higher PV than those with nanoemulsions (*p* < 0.05). These results suggest that both the size of antioxidant carriers and the type of antioxidant play critical roles in controlling lipid oxidation.

In the present study, when the PV results were examined, it was determined that the cookies prepared with a black cumin oil nanoemulsion (BC–C) had the lowest PV during storage. This result was probably due to the high γ-tocopherol content in black cumin. Ceylan et al. (2022)^11^ reported that black cumin oil-loaded nanofibers retarded oxidation in salmon fish fillets cooked with the sous vide technique. Although the PV of the samples containing nanoemulsions (except for the BC-C group) was determined to be higher than the control samples on the 90th day of storage, the upper value of 10 mequiv O_2_ /kg, which is considered the upper value for PV in foods, was not reached. This observation can also be attributed to the fatty acid profile and the specific arrangement of various fatty acids within the triglyceride molecules.^48^

In our study, the oils used in the nanoemulsions exhibited notable antioxidant activities, which likely contributed to the oxidative stability of the cookies. Black cumin oil, for instance, is rich in γ-tocopherol and thymoquinone, compounds known for their strong free radical scavenging capabilities. Sesame oil, another oil used in the study, contains sesamin and sesamol, which have been reported to exhibit significant antioxidant properties. Grape seed oil is also recognized for its high polyphenol content, further supporting its role in delaying lipid oxidation. These antioxidant-rich oils played a critical role in limiting primary oxidation processes during the storage period of the cookies. This observation aligns with findings from Lopez et al. (2024),^48^ who demonstrated that treatments with essential oils effectively reduced PV by over 10% compared to peanut oil.

### 3.9 Thiobarbituric Acid

Lipid oxidation is one of the important factors affecting the shelf life and quality of bakery products. In this respect, oxidation could be evaluated as one of the major factors to reveal the food quality, so TBA analysis is used to detect lipid oxidation in different kinds of foods. In recent studies, it has been shown that innovative approaches, such as nanofiber applications on foods, can effectively limit the rapid lipid oxidation.^53^ TBA results are stated as milligrams of MDA/kg of fat in cookies (Figure 4). TBA values were 1.47–2.51 mg MDA/kg on day 0, 2.01–3.56 mg MDA/kg on day 45, and 2.28–4.13 mg MDA/kg on day 90. Rowayshed et al. (2015) reported that the TBA values of biscuits containing 0.5, 1, 2, and 3% potato peel extract ranged between 0.382 and 0.625, 0.378 and 0.597, 0.376 and 0.578, and 0.376 and 0.550 mg MDA/kg, respectively. In this regard, our results were consistent with the results reported by Rowayshed et al. (2015).^54^ Lipid oxidation is a progressive phenomenon, and the observed increase in the TBA values across the storage period aligns with expectations. However, the stability provided by nanoemulsions, especially in samples with coconut- or sesame oil-loaded nanoemulsions, highlights their potential to delay oxidative changes. This indicates that the type of fat used and its interaction with antioxidants play a significant role in oxidative stability,^47^ ^,^ ^48^

On the 90th day, compared to the zeroth day, TBA values of cookies were increased except for the cookies prepared with coconut oil-loaded nanoemulsion. The control group (C) cookie had the lowest TBA values on day 0 and day 90. The lowest TBA in the control group was expected due to lower unsaturated fat content and higher synthetic antioxidant content. Interestingly, while synthetic antioxidants in the control group demonstrated superior oxidative resistance, nanoemulsions compensated for the absence of synthetic additives in fat-reduced formulations. This suggested that natural antioxidants delivered via nanoemulsions could serve as an alternative to synthetic antioxidants, albeit with limitations in efficiency.

The lowest increase was found in cookies prepared with a sesame-oil-loaded nanoemulsion with 29.4%. The SO-C sample was followed by GS-C (increase: 29.8%), BC-C (increase: 30%), C (increase: 55%), and FC (increase: 107%). When the samples containing nanoemulsions were compared with the FC sample having the same fat amount, the addition of nanoemulsion would delay the oxidation of the cookie oil. As a result, the TBA values of the samples containing nanoemulsions were higher than those of the full-fat-containing control samples on all storage days. This was associated with higher antioxidant content of the control group, as mentioned above. However, TBA values of the samples containing nanoemulsions were lower at the end of 90 days compared to the FC sample with reduced fat and no nanoemulsion.

These findings align with Rowayshed et al. (2015), though differences in formulation and storage conditions may account for variations in TBA values. Future studies could optimize nanoemulsion formulations to enhance their natural antioxidant loading capacity, bridging the gap between natural and synthetic additives, while supporting consumer demand for clean-label products.

## 4 Conclusion

The study aimed to produce cookies with a reduced fat content. Nanoemulsion forms of black cumin, coconut, sesame, and grape seed oils, which are known to have antioxidant properties, were added to the cookie formulation. It was confirmed that nanomaterials of 100–300 nm size were produced. Considering the melting points of the oils, it was determined that the melting temperatures of the oils encapsulated in the nanoemulsion were increased, and their thermal resistance was increased. The most important physical differences in cookies were in the diameter and thickness. While the diameter of the cookies was increased, the thickness was also decreased with the nanoemulsion application. The hardness values of the cookies containing nanoemulsions were lower, and the nanoemulsions produced softer cookies during storage (90 days). Also, nanoemulsions protected cookie oils against oxidation. When the cookies were evaluated in terms of PV, the PV of the cookies prepared with a black cumin oil-loaded nanoemulsion had the lowest value. It was concluded that black cumin oil nanoemulsions could be an alternative to synthetic antioxidants. The nanoemulsions had a significant slowing effect on the TBA increase. The use of nanoemulsions in high-fat-containing foods could be suitable for limiting the TBA value. With this study, it was revealed that nanoemulsions could be used as fat substitutes.

In this study, a novel approach utilizing maltodextrin-based nanoemulsions loaded with natural antioxidant-rich oils was successfully employed as a fat substitute in cookies. This method not only enhanced oxidative stability but also contributed to maintaining desirable sensory attributes throughout the storage period. The combined effects of improved oxidative stability, enhanced texture quality, and potential for clean-label formulation highlight the practical advantages of this innovative method. These findings offer valuable insights for future studies exploring sustainable and effective fat replacement strategies in the food industry.
